# Temporal Reference, Attentional Modulation, and Crossmodal Assimilation

**DOI:** 10.3389/fncom.2018.00039

**Published:** 2018-06-05

**Authors:** Yingqi Wan, Lihan Chen

**Affiliations:** School of Psychological and Cognitive Sciences and Beijing Key Laboratory of Behavior and Mental Health, Peking University, Beijing, China

**Keywords:** temporal window, temporal ventriloquism effect, central tendency effect, assimilation, attention

## Abstract

Crossmodal assimilation effect refers to the prominent phenomenon by which ensemble mean extracted from a sequence of task-irrelevant distractor events, such as auditory intervals, assimilates/biases the perception (such as visual interval) of the subsequent task-relevant target events in another sensory modality. In current experiments, using visual Ternus display, we examined the roles of temporal reference, materialized as the time information accumulated before the onset of target event, as well as the attentional modulation in crossmodal temporal interaction. Specifically, we examined how the global time interval, the mean auditory inter-intervals and the last interval in the auditory sequence assimilate and bias the subsequent percept of visual Ternus motion (element motion vs. group motion). We demonstrated that both the *ensemble (geometric) mean* and the *last interval* in the auditory sequence contribute to bias the percept of visual motion. Longer mean (or last) interval elicited more reports of group motion, whereas the shorter mean (or last) auditory intervals gave rise to more dominant percept of element motion. Importantly, observers have shown dynamic adaptation to the temporal reference of crossmodal assimilation: when the target visual Ternus stimuli were separated by a long gap interval after the preceding sound sequence, the assimilation effect by ensemble mean was reduced. Our findings suggested that crossmodal assimilation relies on a suitable temporal reference on adaptation level, and revealed a general temporal perceptual grouping principle underlying complex audio-visual interactions in everyday dynamic situations.

## Introduction

Multisensory interaction has been traditionally revealed to take place over a narrowed window time—i.e., within a presumed “temporal window” (Meredith et al., 1987; Powers et al., 2009; Vroomen and Keetels, 2010; Wallace and Stevenson, 2014; Gupta and Chen, 2016). For example, paired sound/tactile events presented in temporal proximity to paired visual events can alter the perceived interval between the visual stimuli, and hence bias the perception of visual apparent motion (Keetels and Vroomen, 2008; Chen et al., 2010; Shi et al., 2010). The above illusions have been typically known as temporal ventriloquism (Chen and Vroomen, 2013). Studies on temporal ventriloquism indeed suggested that crossmodal events appearing in temporal proximities have higher probabilities of “correlation” and even “causation” relations (Ernst and Di Luca, 2011; Parise et al., 2012). Based on those relations, sensory events with higher functional priorities (such as “precision” in timing) would calibrate/attract the counterpart events (with lower functional appropriateness) from the other modalities, give rise to successful *multisensory integration*. During the integration, multisensory events within a presumed short time window will largely obey the “assumption of unity,” in which the coherent representation of multiple events become possible when they have been deemed as coming from a common source (Vatakis and Spence, 2007, 2008; Misceo and Taylor, 2011; Chuen and Schutz, 2016; Chen and Spence, 2017). As a result, the effectiveness of crossmodal interaction is enhanced.

However, the presumed “temporal window” for integration has often been violated in many ecological scenarios. Take an example: upon hearing the whistle of a running car behind us, after a decent long delay, we can know exactly what kind of the “car” is approaching and then make prompt avoidance. This indicates that humans can adaptively use the prior knowledge and employ the temporal/spatial information (including environmental cues associated with the sound) to facilitate the perceptual decision. This daily scenario, however, imposes a great challenge for human perception. How are perceptual grouping and correspondences between events achieved when the crossmodal events are separated both in longer temporal ranges and with larger temporal disparities? Moreover, for the longer temporal range, observers have difficulties in memorizing all the events and the processing of the sensory properties (including time information) would probably exceed their working memory capacities (Cowan, 2001; Klemen et al., 2009; Klemen and Chambers, 2012; Cohen et al., 2016). Therefore, the efficiency of crossmodal interaction will be reduced accordingly. The complex timing scenario as well as the challenge for time cognition also stems from the variance of the multiple time intervals. In short temporal range (such as around 2 s), human observers could discriminate the short temporal intervals when the coefficient of variance (i.e., “CV,” the ratio of the interval deviation to its baseline value) is less than 0.3. The discrimination ability is greatly reduced when the CV is above 0.3 (Allan, 1974; Getty, 1975; Penney et al., 2000).

To cope with the above constraints, human observers adopt one of the efficient perceptual strategies—“ensemble coding” to process the mean properties of multiple events. For example, people can extract the mean rhythm of a given sound sequence and use this information to allocate visual attention and facilitate the detection of target events (Miller et al., 2013). Recent studies have shown that this averaging process is highly dependent on the temporal reference. The temporal reference included the generally global time interval before the onset of target event(s), the variabilities of the multiple intervals and the critical information of the last interval (Jones and McAuley, 2005; Acerbi et al., 2012; Cardinal, 2015; Karaminis et al., 2016). One compelling example is the central tendency effect within the broader framework of Bayesian optimization (Jazayeri and Shadlen, 2010; Shi et al., 2013; Shi and Burr, 2016; Roach et al., 2017), whereby incorporating the mean of the statistical distribution in the estimation would assimilate the estimates toward the mean (Jazayeri and Shadlen, 2010; Burr et al., 2013; Karaminis et al., 2016). For example, the estimation of a target property, such as the duration of an event, is assimilated toward to the mean duration of previously encountered target events (i.e., event history) (Nakajima et al., 1992; Burr et al., 2013; Shi et al., 2013; Roach et al., 2017). The central tendency effect indicates that human observers exploit predictive coding using the averaged sensory properties (Shi and Burr, 2016). The predictive coding framework states that the brain produces a Bayesian estimate of the environment (Friston, 2010). A strong mismatch between the prediction and the actual sensory input leads to an update of the internal model, and could trigger observable changes in perceptual decision. During this updating, attentional process can be considered as a form of predictive coding to establish an expectation of the moments in time until the task-relevant, to be integrated stimulus inputs arrive (Klemen and Chambers, 2012). On the other hand, the temporal reference (including temporal window) for crossmodal interaction is flexible by perceptual training (Powers et al., 2009, 2012), repeated exposure (adaptation) to the sensory stimuli (Mégevand et al., 2013), or recalibration process through experience (Sugano et al., 2010, 2012, 2016; Bruns and Röder, 2015; Habets et al., 2017). The flexibility of temporal window has also been shown to be shaped by the individual differences (Hillock et al., 2011; Stevenson et al., 2012, 2014; Lewkowicz and Flom, 2014; Chen et al., 2016; Hillock-Dunn et al., 2016).

Time perception is intrinsically related with attention and memory (Block and Gruber, 2014). Attention has been revealed to act as an essential cognitive faculty in integrating information in the multisensory mind (Duncan et al., 1997; Talsma et al., 2007, 2010; Donohue et al., 2011, 2015; Tang et al., 2016). (Selective) attention improves the efficiency of pooling task-relevant information - multiple (complex) properties (Buchan and Munhall, 2011; Li et al., 2016). Withdrawing attention has been shown in other tasks/paradigms to degrade the representation of individual sensory properties (Alsius et al., 2005, 2014). In the central tendency effect, observers processed task-relevant sensory properties to obtain the subsequent perceptual decision. However, whether/how attentional modulation would deplete the limited attentional resources for ensemble coding and hence play a role in the crossmodal assimilation, has not been empirically examined.

Therefore, in the present study, we aimed to examine how the temporal reference and the attentional processing would affect the crossmodal assimilation. We adopted “temporal ventriloquism effect” with visual Ternus display. We investigated how the temporal configurations between an auditory sequence (with multiple inter-intervals) and the visual Ternus display (with one interval) modulate the visual apparent-motion percepts. Ternus display can elicit two distinct percepts of visual apparent motion: “element” motion or “group” motion, determined by the visual inter-stimulus-interval (ISI_V_) between the two display frames (with other stimulus settings being fixed). Element motion is typically observed with short ISI_V_ (e.g., of 50 ms), and group motion with long ISI_V_ (e.g., of 230ms) (Ternus, 1926; Shi et al., 2010) (see Supplement 1 for visual animation of Ternus display). Previously we have shown that when two beeps were presented in temporal proximity to, or synchronously with, the two visual frames respectively, the beeps can systematically bias the transitional threshold of visual apparent motion (Shi et al., 2010). Here we extended the Ternus temporal ventriloquism paradigm to investigate the temporal crossmodal ensemble coding. We implemented five experiments to address this issue. Experiments 1 and 2 examined the role of temporal window- interval gap between the offset of sound sequence and the onset of target Ternus display, to show the temporal constraints of central tendency effect. Experiment 3 compared the central tendency effect with the recency effect, by manipulating both the mean auditory interval and the last auditory interval. In Experiment 4, we fixed the last interval to be equal to the transitional threshold of perceiving element vs. group motion in the pretest, and manipulated the mean auditory inter-interval to show a genuine central tendency effect during crossmodal assimilation. In Experiment 5, we implemented dual-tasks and asked observers to perform the visual Ternus task while fulfilling a concurrent task of counting oddball sounds. Overall, the current results revealed that crossmodal central tendency effect is subject to the temporal reference (including the length of global time interval, the mean interval and the last interval for a given sound sequence) but less dependent on attentional modulation.

## Materials and methods

### Participants

A total of 60 participants (14, 13, 7, 12, 14 in Experiments 1–5), ages ranging from 18 to 33 years, took part in the main experiments. A *post-hoc* power estimation has shown the statistical powers are generally approaching or above 0.8 for the given sample sizes. All observers had normal or corrected-to-normal vision and reported normal hearing. The experiments were performed in compliance with the institutional guidelines set by the Academic Affairs Committee, School of Psychological and Cognitive Sciences, Peking University. The protocol was approved by the Committee for Protecting Human and Animal Subjects, School of Psychological and Cognitive Sciences, Peking University. All participants gave written informed consent in accordance with the Declaration of Helsinki, and were paid for their time on a basis of 40 CNY/hour, i.e., 6.3 US dollars/hour.

### Apparatus and stimuli

**Figure 1:**
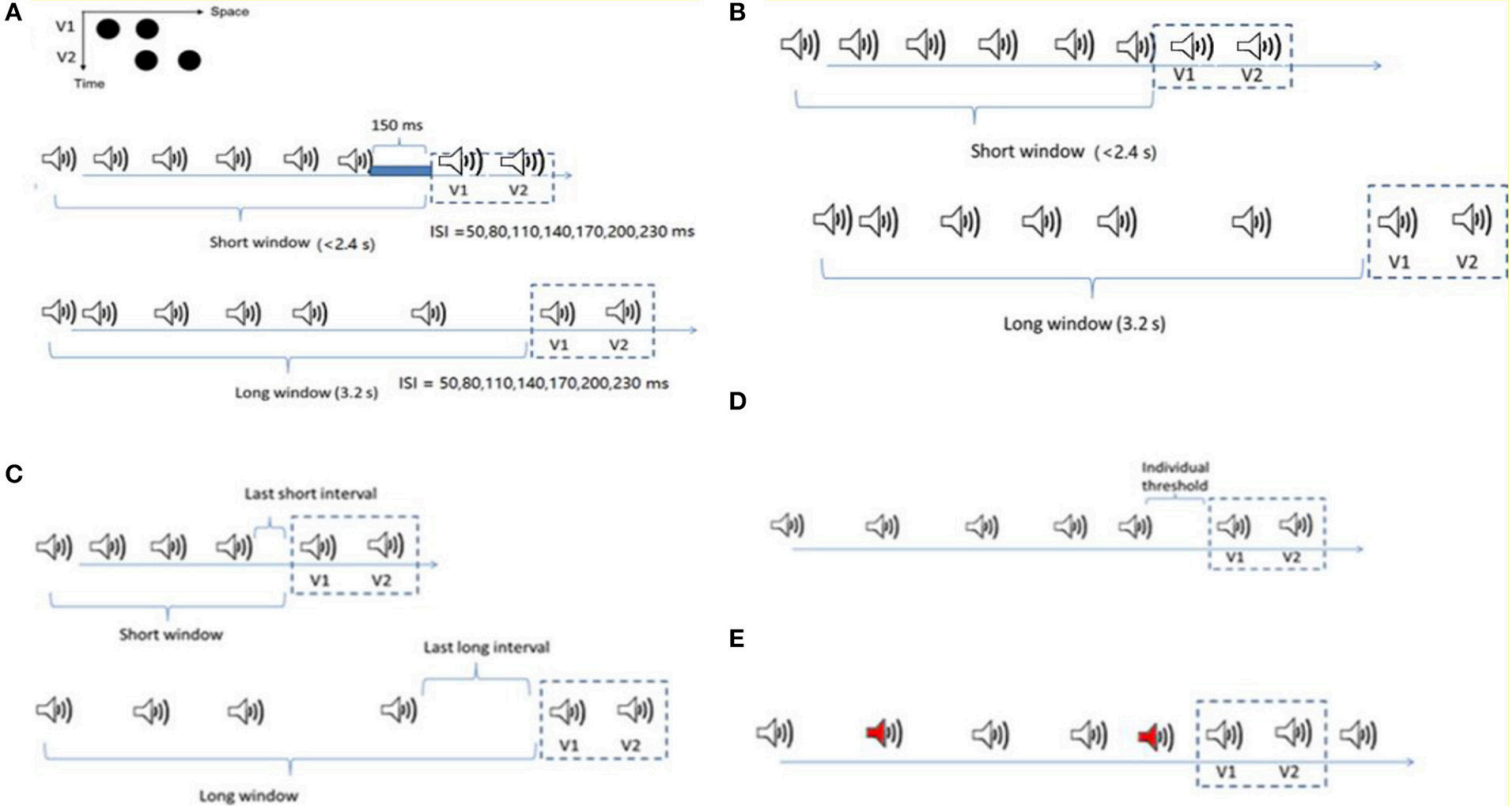
Stimuli configurations for the four experiments. **(A)** Ternus display: two alternative motion percepts of the Ternus display—“element” motion for the short ISIs, with the middle black dot perceived as remaining static while the outer dots are perceived to move from one side to the other. “Group” motion for long ISIs, with the two dots perceived as moving in tandem. The auditory sequence consisted of 6 to 8 beeps (with 7 beeps as the most frequent cases). The Ternus display, with 50 to 230ms interval between the two frames, was followed by a blank interval of 150ms to the offset of the last beep in the short time window condition (the total interval length from the onset of the first beep to the onset of the first visual Ternus frame was less than 2.4 s), and 3.2 s in the long time window condition. In both the short and long window conditions, two beeps were synchronously paired with two visual Ternus frames. **(B)** The configuration was nearly the same as in **(A)**, but for the short window condition, the two frames followed immediately with the last beep. **(C)** The competition between the mean interval in temporal window and the last auditory interval upon the visual Ternus motion. The mean auditory inter-intervals/last auditory intervals could be longer (transition threshold + 70ms) or shorter (transition threshold −70ms) than the threshold between the element—and group—motion percept. The lengths for both short and long time windows were the same as in **(A)**. **(D)** Two types of auditory sequences with five auditory intervals were composed: one with its geometric mean 70ms shorter than the transition threshold of the visual Ternus motion (“Short” condition), and the other with its geometric mean 70ms longer than the transitional threshold (“Long” condition). The last auditory interval before the onset of Ternus display was fixed at the individual “transitional threshold” for both sequences. **(E)** The configuration was similar as in C but the sound sequence had up to two oddball sounds (500 Hz, here we showed two oddball sounds with red labels). The remaining regular sounds were of 1,000 Hz (including the two beeps synchronous with the two visual frames).

The experiments were conducted in a dimly lit (luminance: 0.09 cd/m^2^) room. Visual stimuli were presented at the center of a 22-inch CRT monitor (FD 225P) at a screen resolution of 1024 × 768 pixels and a refresh rate of 100 Hz. Viewing distance was 57 cm, maintained by using a chin rest. A Ternus display consisted of two stimulus frames, each containing two black discs (l0.30 cd/m^2^; disc diameter and separation between discs: 1.6° and 3° of visual angle, respectively) presented on a gray background (16.3 cd/m^2^). The two frames shared one element location at the center of the monitor, while containing two other elements located at horizontally opposite positions relative to the center (see Figure 1A). Each frame was presented for 30ms; the inter-stimulus interval (ISI_V_) between the two frames was randomly selected from the range of 50–230ms, with a step size of 30ms.

Mono sound beeps (1,000 Hz pure tone, 65 dB SPL, 30ms, except in Experiment 5 where pure tones with pitches of either 1,000 Hz or 500 Hz were given) were generated and delivered via an M-Audio card (Delta 1010) to a headset (Philips, SHM1900). No ramps were applied to modulate the shape of the tone envelope. To ensure accurate timing of the auditory and visual stimuli, the duration of the visual stimuli and the synchronization of the auditory and visual stimuli were controlled via the monitor's vertical synchronization pulses. The experimental program was written with Matlab (Mathworks Inc.) and the Psychophysics Toolbox (Brainard, 1997; Kleiner et al., 2007).

### Experimental design

#### Practice

Prior to the formal experiment, participants were familiarized with Ternus displays of either typical “element motion” (with an interval of 50ms) or “group motion” (with an interval of 260ms) in a practice block. They were asked to discriminate the two types of apparent motion by pressing the left or the right mouse button, respectively. The mapping between response button and type of motion was counterbalanced across participants. During practice, when an incorrect response was made, immediate feedback appeared on the screen showing the correct response (i.e., element or group motion). The practice session continued until the participant reached a mean accuracy of 95%. All participants achieved this within 120 trials.

#### Pre-test

For each participant, the transition threshold between element and group motion was determined in a pre-test session. A trial began with the presentation of a central fixation cross lasting 300 to 500ms. After a blank screen of 600ms, the two Ternus frames were presented, synchronized with two auditory tones [i.e., baseline: ISIV(isual) = ISIA(uditory)]; this was followed by a blank screen of 300 to 500ms, prior to a screen with a question mark prompting the participant to make a two-alternative forced-choice response indicating the type of perceived motion (element or group motion). The ISI_V_ between the two visual frames was randomly selected from one of the following seven intervals: 50, 80, 110, 140, 170, 200, and 230ms. There were 40 trials for each level of ISI_V_, counterbalanced with left- and rightward apparent motion. The presentation order of the trials was randomized for each participant. Participants performed a total of 280 trials, divided into 4 blocks of 70 trials each. After completing the pre-test, the proportions of the group motion responses across seven intervals were fitted to the psychometric curve using a logistic function (Treutwein and Strasburger, 1999; Wichmann and Hill, 2001). The transitional threshold, that is, the point of subjective equality (PSE) at which the participant was likely to report the two motion percepts equally, was calculated by estimating 50% of reporting of group motion on the fitted curve. The just noticeable difference (JND), an indicator of the sensitivity of apparent motion discrimination, was calculated as half of the difference between the lower (25%) and upper (75%) bounds of the thresholds from the psychometric curve.

#### Main experiments

In the main experiments, the procedure for presenting visual stimuli was the same as in the pre-test session, except that prior to the occurrence of two Ternus-display frames, an auditory sequence consisting a variable number of 6–8 beeps was presented (see below for the details of the onset of Ternus-display frames relative to that of the auditory sequence). A trial began with the presentation of a central fixation marker, randomly for 300 to 500ms. After a 600-ms blank interval, the auditory train and the visual Ternus frames were presented (see Figure 1A), followed sequentially by a blank screen of 300 to 500ms and a screen with a question mark at the screen center prompting participants to indicate the type of motion they had perceived: element vs. group motion (non-speeded response). During the experiment, observers were simply asked to indicate the type of visual motion (“element” or “group” motion) that they perceived, while ignoring the beeps. After the response, the next trial started following a random inter-trial interval of 500 to 700ms.

In Experiment 1, the visual Ternus frames were preceded by an auditory sequence of 6–8 beeps with the geometric mean of inter-stimulus interval [ISIA(uditory), i.e., ISI_A_], manipulated to be 70ms shorter than, or 70ms longer than the transition threshold estimated in the pre-test. The [ISIV(isual), i.e., ISI_V_] between the two visual Ternus frames was randomly selected from one of the following seven intervals: 50, 80, 110, 140, 170, 200, and 230ms. The total auditory sequence consisted of 6–8 beeps. Visual Ternus frames were presented on most of all trials (672 trials in total) following the last beep; the remaining were catch trials (72 trials) in which the frames were inset in the sound sequence to break up anticipatory processes. For the short time window of the auditory sequence, the time interval from the onset of the first beep to the onset of the first visual frame was less than 2.4 s, and the gap interval between the offset of the last beep and the onset of the first Ternus frame was 150ms. For the long time window, the total interval from the onset of the sound to the first visual frame was 3.2 s. In both the short and long window conditions, two beeps were synchronously paired with two visual Ternus frames. All the trials were randomized and organized in 12 blocks (62 trials for each block).

In Experiment 2, the settings were the same as in Experiment 1, except for the condition: the visual frames were following immediately with the offset of the last beep.

In Experiment 3, we introduced two factors of interval modulations: the mean interval of temporal window and the last auditory interval. The mean auditory inter-intervals and the last auditory intervals could be larger (transition threshold + 70ms) or shorter (transition threshold −70ms) than the threshold between the element- and group- motion percept. Therefore, there were four combinations of the “interval” conditions: both the mean interval and the last interval were shorter (i.e., “MeanSLastS”); the mean interval was shorter but the last interval was longer (“MeanSLastL”); the mean was longer but the last interval was shorter (“MeanLLastS”); and both the mean interval and the last interval were longer (“MeanLLastL”). The onset of the two visual Ternus frames (30ms) was accompanied by a (30-ms) auditory beep (i.e., ISI_V_ = ISI_A_).

In Experiment 4 we compared two auditory sequences: one with its geometric mean 70ms shorter than the transition threshold of the visual Ternus motion (hereafter the “Short” condition), and the other with its geometric mean 70ms longer than the transitional threshold (hereafter the “Long” condition). Instead of randomization of the five auditory intervals (excluding the final synchronous auditory interval with the visual Ternus interval), the last auditory interval before the onset of Ternus display was fixed at the “transitional threshold” for both sequences. The rest four intervals were chosen randomly such that the coefficient of variance (CV) of the auditory sequence was in the range between 0.1 and 0.2, which is the normal range of CV for human observers (Allan, 1974; Getty, 1975; Penney et al., 2000). By this manipulation, we expected to minimize the influence of the potential recency effect caused by the last auditory interval. The audiovisual Ternus frames were appended at the end of these sequences for 85.7% trials (with 672 trials out of 784 trials), in which the Ternus display appeared at the end of the sound sequence (the “onset” of first visual frame was synchronized with 6th beep). The remaining were 112 catch trials, in which 56 trials had the Ternus displays at the beginning of the sound sequence (i.e., the “onset” of the first visual frame was synchronized with the second beep), and the rest 56 trials at middle temporal locations (i.e., the “onset” of the first visual frame was synchronized with the 4th beep). Those catch trials were used to avoid potential anticipatory attending to the visual events appearing at the end of the sound sequence. The total 784 trials were randomized and organized in 14 blocks, with each of 56 trials.

In Experiment 5, we used three types of auditory sequences, in which the mean auditory interval was either *shorter than, equal to* or *longer than* the individual transitional threshold of Ternus motion. The auditory sequence consisted of 8 to 10 beeps, including those accompanying the two visual Ternus frames, with the latter being inserted mainly at the 6th−7th positions (504 trials), and followed by 0–2 beeps (number selected at random), to minimize expectations for the onset of the visual Ternus frames. Two of the beeps (the 6th and the 7th) were synchronously paired with two visual Ternus frames which were separated by a visual ISI (ISI_V_) that varied from 50 to 230ms (for the critical beeps, ISI_V_ = ISI_A_). There were up to two oddball tones (500 Hz) in the sound sequence, while the remaining regular sounds were of 1,000 Hz (including the two beeps synchronous with the two visual frames). Participants completed a dual-task in which they not only made discriminations of the Ternus display (“element motion” vs. “group motion”) but also reported the number of oddball sounds (0–2) (Figure 1).

## Results

### Experiment 1: the effect of short temporal window (with a temporal gap between auditory sequence and visual ternus) vs. long temporal window

**Figure 2:**
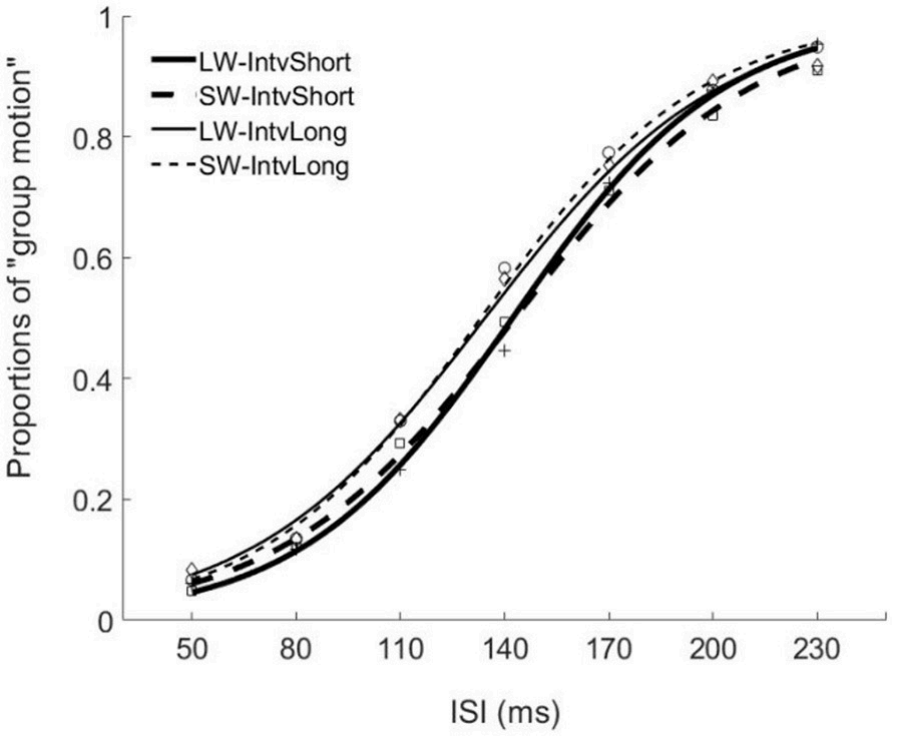
Psychometric curves for Experiment 1. Mean proportions of group-motion responses were plotted as a function of the probe visual interval (ISIv), and fitted psychometric curves, were plotted for the auditory sequences with the different lengths of temporal windows and with different (geometric) mean intervals relative to the individual transition thresholds. SW-IntvLong, Short window with long mean auditory inter-interval; SW-IntvShort, Short window with short mean auditory inter-interval; LW-IntvLong, Long window with long mean auditory inter-interval. LW-IntvShort, long window with short mean auditory inter-interval.

The PSEs for the short window and long window were 149.4 (±5.6, standard error)ms and 141.2 (±4.8)ms. The main effect of temporal window was significant, *F*_(1, 13)_ = 6.878, *p* = 0.021, $\eta_{g}^{2}\;=\;0.346$. The PSEs for short interval and long interval were 145.5(±5.2)ms and 145.0 (±4.8)ms, the main effect of mean interval was not significant, *F*_(1, 13)_ = 0.120, *p* = 0.735, $\eta_{g}^{2\;}=\;0.009.$ The interaction effect between factors of window and interval was not significant, *F*_(1, 13)_ = 1.033, *p* = 0.328, $\eta_{g}^{2}=0.074.$ For the JNDs, both the main effects of temporal window and mean interval were not significant, *F*_(1, 13)_ = 3.419, *p* = 0.087, $\eta_{g}^{2}=0.208\;$and *F*_(1, 13)_ = 0.089, *p* = 0.770, $\eta_{g}^{2}=0.007.$ And the interaction effect between the two factors was not significant, *F*_(1, 13)_ = 2.863, *p* = 0.114, $\eta_{g}^{2}=0.180\;$(Figures 2, **4**).

### Experiment 2: the effect of short temporal window (without a gap between auditory sequence and visual ternus) vs. long temporal window

The PSEs for the short window and long window were 168.7 (±6.2)ms and 156.2 (±5.7). The PSE for short window was larger than the one in long window, *F*_(1, 12)_ = 20.860, *p* = 0.001, $\eta_{g}^{2}=0.635$. The PSEs for short interval and long interval were 163.8 (±6.0)ms and 161.0 (±5.8), the main effect of mean interval was not significant, *F*_(1, 12)_ = 1.869, *p* = 0.197, $\eta_{g}^{2}=0.135.$ Importantly, the interaction effect between factors of window and interval was significant, *F*_(1, 12)_ = 5.090, *p* = 0.044, $\eta_{g}^{2}=0.298.$ Further simple effect analyses showed that for short interval, the PSE in short window (172.7 ± 7.3ms) was larger than the one (154.9 ± 5.3ms) in long window, *p* = 0.001. For long interval, the PSE in short window (164.7 ± 5.5ms) was larger than the one (157.3 ± 6.4ms) in long window, *p* = 0.034. On the other hand, for the short window, the PSE in short interval (172.7 ± 7.3ms) was larger than the one in long interval (164.7 ± 5.5ms), *p* = 0.044. However, for the long window, the PSEs are equal in both intervals (154.9 vs. 157.3ms for short and long intervals), *p* = 0.377.

**Figure 3:**
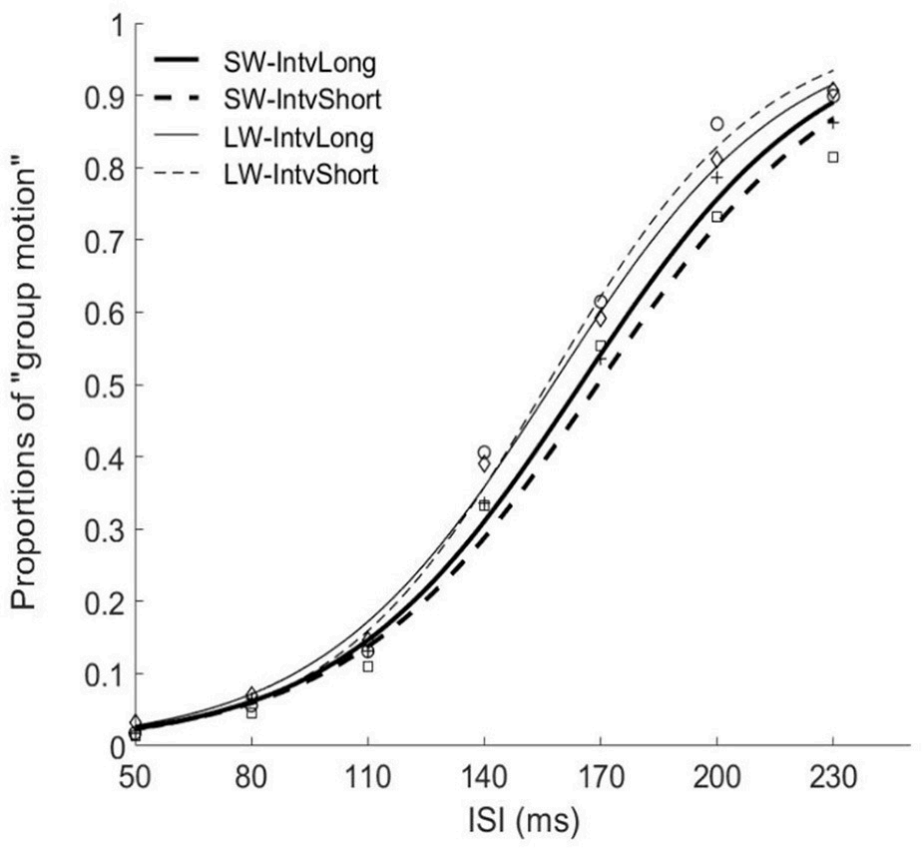
Psychometric curves for Experiment 2. SW-IntvLong, Short window with long mean auditory inter-interval; SW-IntvShort, Short window with short mean auditory inter-interval; LW-IntvLong, Long window with long mean auditory inter-interval. LW-IntvShort, long window with short mean auditory inter-interval.

**Figure 4:**
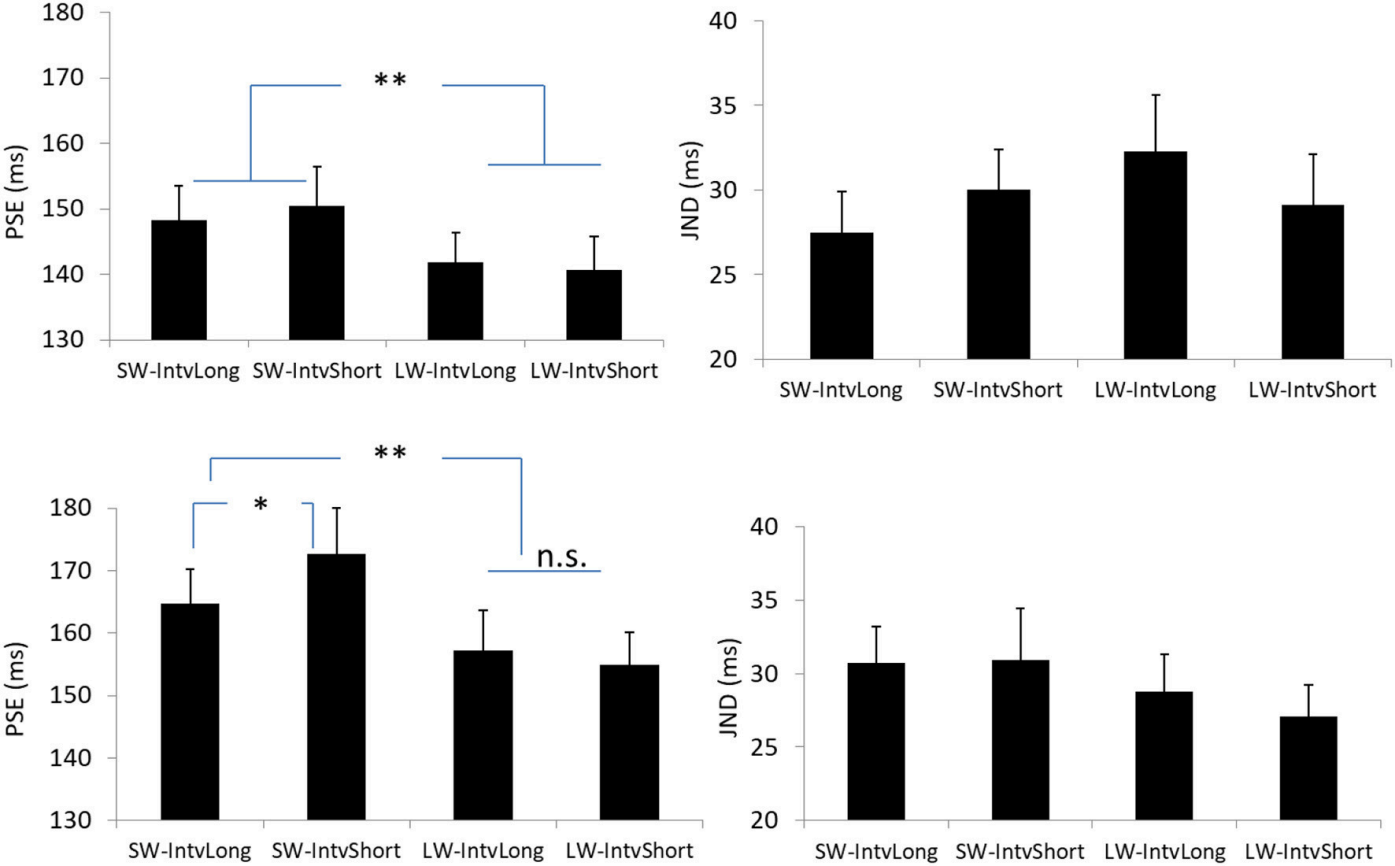
Plotted bars for PSE (point of subjective equality) and JND (just noticeable difference) for Experiments 1 **(Upper)** and 2 **(Down)**. *p < 0.05, **p < 0.01, n.s. not significant.

For the JNDs, both the main effects of temporal window and mean interval were not significant [*F*_(1, 12)_ = 2.479, *p* = 0.141, $\eta_{g}^{2}=0.171\;$and *F*_(1, 12)_ = 0.282, *p* = 0.605, $\eta_{g}^{2}=0.023].$ The interaction effect between the two factors was not significant, *F*_(1, 12)_ = 0.408, *p* = 0.535, $\eta_{g}^{2}=0.033$ (Figures 3, 4).

### Experiment 3: central tendency effect vs. last interval

**Figure 5:**
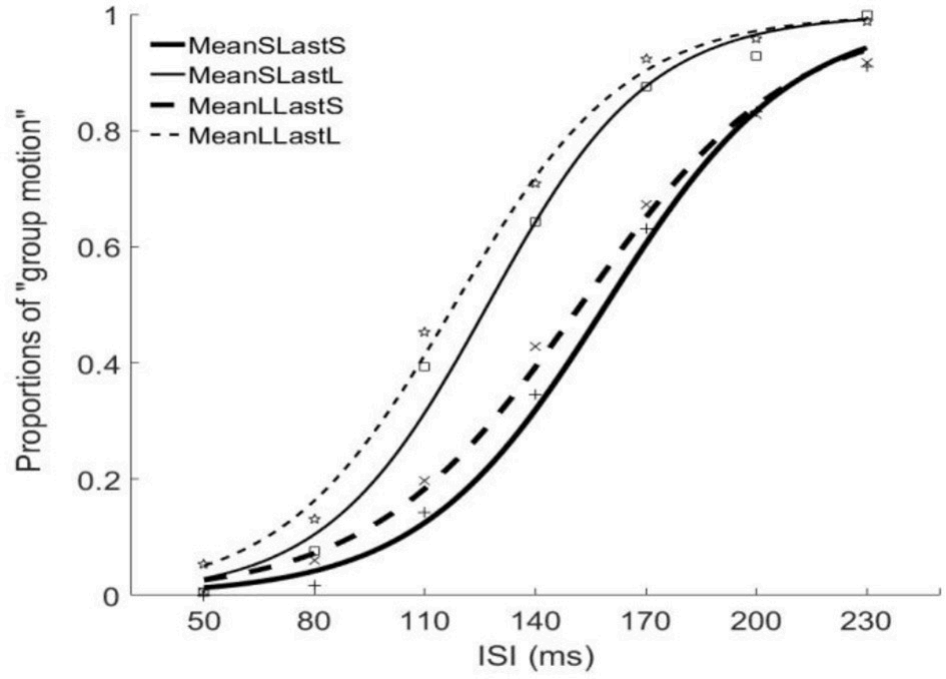
Psychometric curves for Experiment 3. MeanSLastS (bold solid line), Mean short interval with long last auditory interval; MeanSLastL(thin solid line), Mean short interval with short last auditory interval; MeanLLastS(bold dashed line), Mean long interval with short last auditory interval; MeanLLastL(thin dashed line), Mean long interval with long last auditory interval.

**Figure 6:**
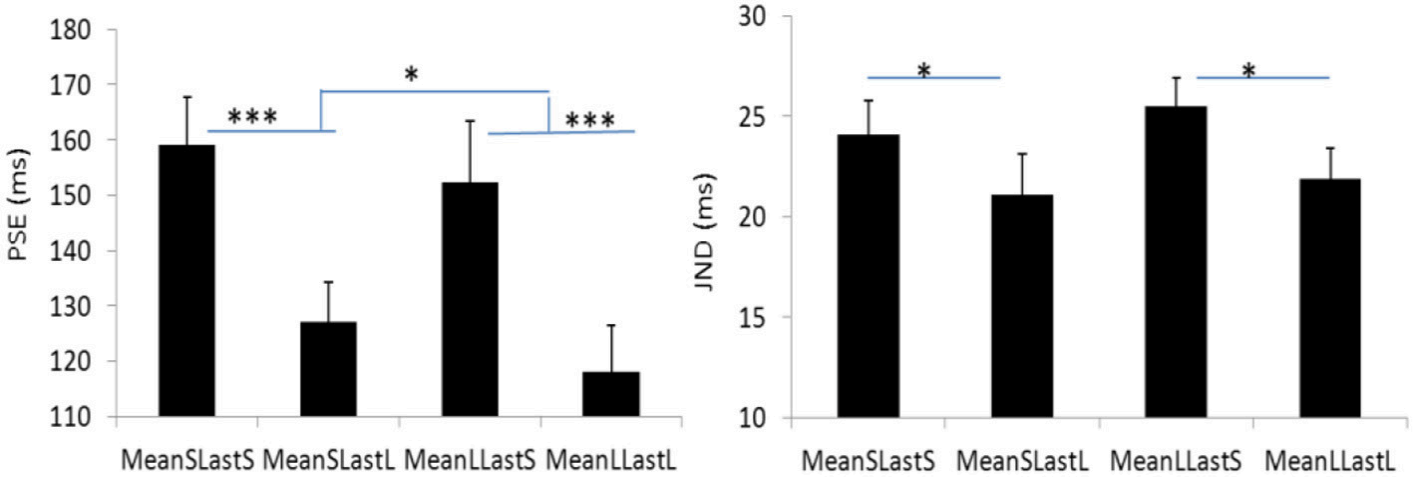
Plotted bars for PSE (point of subjective equality) and JND (just noticeable difference) for Experiment 3. *p < 0.05, **p < 0.01, and ***p < 0.001.

The PSEs for the short mean interval and long mean interval were 143.2 (±7.4)ms and 135.3(±9.5). The main effect of mean interval was significant, *F*_(1, 6)_ = 9.070, *p* = 0.024, $\eta_{g}^{2}=0.602$. The PSEs for short last interval and long last interval were 155.8 (±9.7)ms and 122.6 (±7.5)ms, respectively. The main effect of last interval was significant, *F*_(1, 6)_ = 65.970, *p* = 0.000, $\eta_{g}^{2}=0.917.$ The interaction effect between factors of mean interval and last interval was not significant, *F*_(1, 6)_ = 0.195, *p* = 0.674, $\eta_{g}^{2}=0.031.$ For the JNDs, the JND in short last interval (24.8 ± 1.3ms) was larger than the one in long last interval (21.5 ± 1.6ms), *F*_(1, 6)_ = 11.590, *p* = 0.014, $\eta_{g}^{2}=0.659.$ However, the main effect of mean interval was not significant, *F*_(1, 6)_ = 0.762, *p* = 0.416, $\eta_{g}^{2}=0.113.$ The interaction effect between the two factors was also not significant, *F*_(1, 6)_ = 0.109, *p* = 0.753, $\eta_{g}^{2}=0.018.$ (Figures 5, 6).

### Experiment 4: central tendency effect but with the last interval fixed

**Figure 7:**
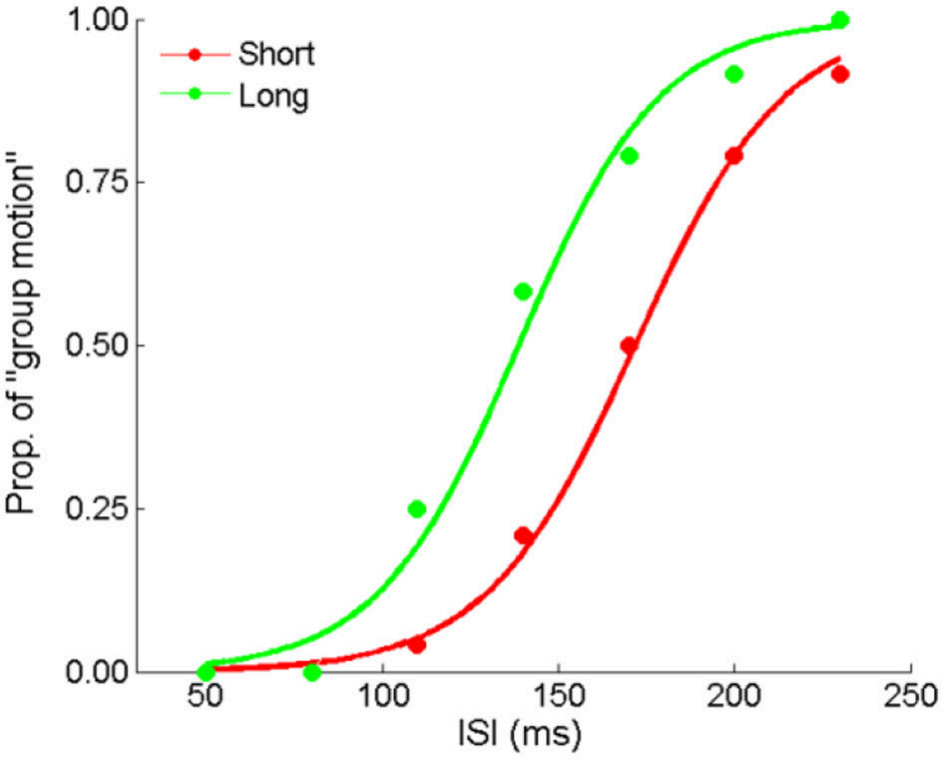
Mean proportions of group-motion responses from a typical participant are plotted against the probe visual interval (ISIv), and fitted psychometric curves for the two geometric mean conditions: the “Short” sequence (with the smaller geometric mean) and “Long” sequence (with the larger geometric mean) in Experiment 4.

Here we made formal manipulation by keeping the last interval fixed for the “Short” and “Long” auditory sequences. Figure 7 depicts the responses from a typical participant. The PSEs were 153.1 (±7.3), 137.9 (±9.1) for the “Short” and “Long” conditions, *t*_(11)_ = 3.640, *p* < 0.01. Participants perceived more dominant percept of Element motion in the “Short” condition than in the “Long” condition, consistent with the findings of the previous experiments. That is, the auditory ensemble mean still assimilated visual Ternus apparent motion when the last interval of the auditory sequence was fixed. Therefore, the audiovisual interactions we found were unlikely only due to the recency effect.

### Experiment 5: central tendency effect with attentional modulation

The PSEs for the *baseline, short, equal*, and *long* intervals were 135.9(±3.3), 171.1(±8.9), 151.5 (±9.5), and142.1(±7.4)ms, the main effect of mean interval was significant, *F*_(2, 39)_ = 9.020, *p* < 0.001, $\eta_{g}^{2}=0.410$. Bonferroni corrected comparison showed that the PSE for baseline was smaller than the one in short condition, *p* = 0.014. PSE for short interval condition was larger than the one in equal condition, *p* = 0.01; and the PSE for short interval was also larger than the ones in the equal and long intervals, *p* = 0.019 and *p* = 0.010. However, the PSEs were equal for both “equal” and “long” conditions, *p* = 0.411. The PSEs were equal for both “baseline” and “equal” condition, *p* = 0.603, and were equal between “baseline” and “long” conditions, *p* = 1.

**Figure 8:**
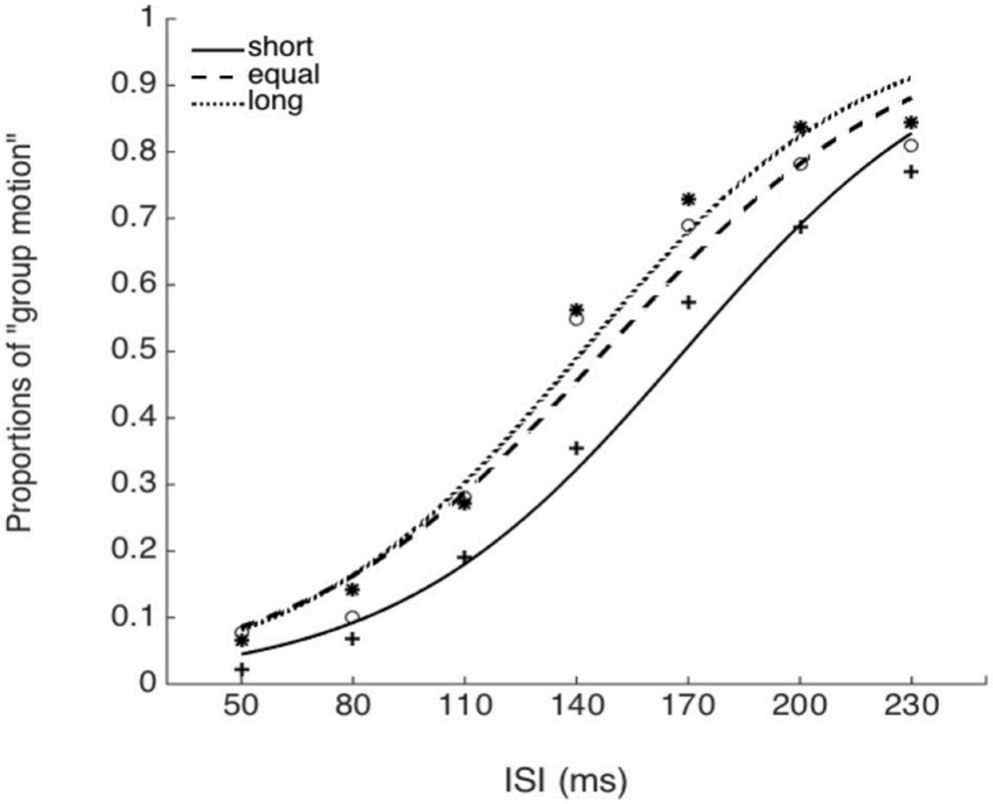
Psychometric curves for Experiment 5. Short (solid line), the mean auditory inter-interval is shorter than the PSE for visual Ternus motion; Equal (dashed line), the mean auditory inter-interval is equal to the PSE for visual Ternus motion; Long (dotted line), the mean auditory inter-interval is longer than the PSE for visual Ternus motion. The PSE (“transitional threshold”) of Ternus motion was established by a pre-test for each individual.

**Figure 9:**
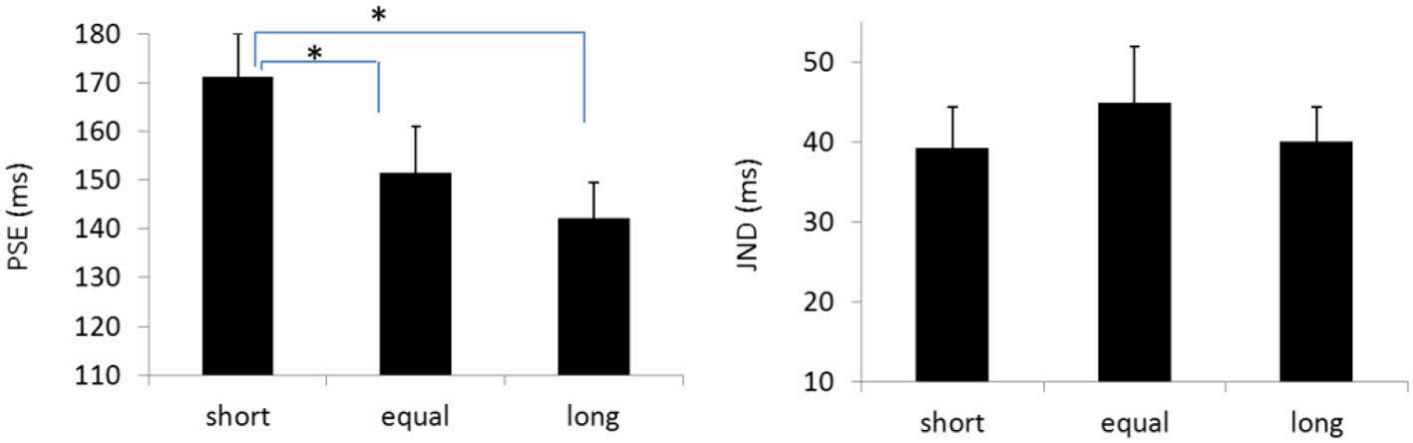
Plotted bars for PSE (point of subjective equality) and JND (just noticeable difference) for Experiments 5, *p < 0.05.

The JNDs for the *baseline, short, equal*, and *long* intervals were 32.2 (±3.7), 39.3 (±5.1), 44.9 (±7.0), and 40.0 (±4.4)ms, respectively. The main effect of mean interval was not significant, *F*_(3, 39)_ = 2.741, *p* = 0.056, $\eta_{g}^{2}=0.174\;$(Figures 8, 9).

The mean correct rate for reporting the number of oddball sounds was 83.0 ± 3.1%, one sample *T*-test with comparison of 50% showed the correct rate was above the chance level [*t*_(13)_ = 10.518, *p* = 9.984 × 10^−8^].

## Discussion

Central tendency, the tendency of judgments of quantitative properties (lengths, durations etc) for given stimuli to gravitate toward their mean, is one of the most robust perceptual effects. The present study has shown that perceptual averaging of temporal property—auditory intervals, assimilates the visual interval between the two Ternus-display frames, and biases the perception of Ternus apparent motion (either to be dominant “element motion” or dominant “group motion”). This finding is consistent with the large body of literature on temporal-context and central tendency effects, within the broader framework of Bayesian optimization (Jazayeri and Shadlen, 2010; Shi et al., 2013; Roach et al., 2017), whereby incorporating the mean of the statistical distribution in the estimation would assimilate the estimates toward the mean—known as “central tendency effect” (Jazayeri and Shadlen, 2010; Burr et al., 2013; Karaminis et al., 2016).

By using the paradigm of temporal ventriloquism and the probe of visual Ternus display (Chen et al., 2010; Shi et al., 2010; Chen and Vroomen, 2013), we have previously shown that the auditory capture effect upon the visual events, in which the perceived visual interval was biased by concurrently presented auditory events. Observers tended to report the illusory visual (apparent motion) percepts with the concurrent presence of auditory beeps. However, the visual-auditory integration effect is subject to the temporal reference, i.e., the time interval between the critical visual probe and the sound sequence, the mean auditory interval and the critical interval between the last auditory stimulus and the onset of visual events. In our current setting, when the total time interval between the onset of auditory signal and the onset of visual events was above 3 s (3.2 s), it gave rise to a diminished central tendency effect. On the contrary, when this time interval was less than 2.4 s, the shortened time reference increased the likelihood of central tendency effect—materialized in the effect of “geometric” perceptual averaging for auditory intervals upon the visual Ternus motion. These findings indicate a general temporal framework of crossmodal integration. As stated in a theoretical construct of temporal perception, known as the “subjective present”—a mechanism of temporal integration binds successive events into perceptual units of 3 s duration (Pöppel, 1997). Such a temporal integration, which is automatic and pre-semantic, is also operative in movement control and other cognitive activities. In this hierarchical temporal model, the temporal reference for temporal binding could be extended but limited within 3 s, together with a memory store (Pöppel, 1997; Pöppel and Bao, 2014). When the framework exceeds 3 s, the integration of the preceding auditory interval information could be decayed, which hence makes the auditory assimilation effect reduced.

Interestingly, even with the presumed short temporal window (within 2.4 s), by inserting a short temporal gap (150ms) between the offset of the very last beep and the onset of the first visual frame, we found the central tendency effect was reduced, and the effect was similar to the results in long temporal window condition (3.2 s). This finding suggests that the “imminent” and most recent (“immediate”) temporal gap before the target visual event is critical for the development of the central tendency effect. This inference is further substantiated by the results from Experiments 2 and 3. In Experiment 2, with the configuration of “short window,” we eliminated the short gap (150ms) between the offset of the last beep and the onset of the visual frames. We found that the central tendency effect (short mean interval. vs. long mean interval) reappeared, though it still remains absent in the condition of “long window.” Moreover, in Experiment 3, we further found that the assimilation effect of the last interval dominates that of the mean auditory interval. This indicates that the last auditory interval wins the competition over the mean interval in driving the crossmodal assimilation.

However, the central tendency effect was less dependent on attentional modulation. Using the dual-tasks of reporting the percept of visual Ternus motion and the number of oddball stimuli [i.e., identifying the number of 500 Hz beep(s) within a sound sequence], we again found the central tendency effect was robust. The observers have invested large attentional resources to obtain the decent performance of counting the oddball sounds. Nevertheless, the performance of crossmodal assimilation effect still survived. Therefore, the central tendency effect as shown in the present study, has demonstrated its automatic and attentional-less demanding nature during crossmodal interaction (Vroomen et al., 2001; Wahn and Konig, 2015).

The current study has some limitations. Indeed, the temporal reference before the target visual Ternus display includes intervals composed by stimuli with different configurations. The auditory sequence was organized by filled-durations with multiple beeps, and there was a transition of intra-modal perceptual grouping (with sounds) to cross-modal grouping when the last beep was followed by the onset of the first visual Ternus frame (with audiovisual events) (Burr et al., 2013). However, the “critical” time window for multisensory integration was presented as an “empty interval” between the two visual frames. Therefore, the visual probe we adopted in current experimental paradigm might restrict the manifestation of assimilation effect, which was probably due to the differential timing sensitivities to the “filled-duration” in auditory sequence vs. “empty-duration” in the visual probe (Rammsayer and Lima, 1991; Grondin, 1993; Rammsayer, 2010). Moreover, the temporal window, as shown in the auditory sequence, covaried with the mean ISIs (mean auditory intervals). This potential confound remains even although we have manipulated the comparisons of durations between the mean ISIs and the critical interval between the two visual frames (Experiments 1, 2, 3, and 5), and tried to tease apart the “central tendency effect” vs. “recency effect” by fixing the last intervals. Further research is needed to elucidate this point.

Taken together, the current study has shown that crossmodal assimilation in temporal domain is shaped by the temporal reference, in which the observers use the temporal information by dynamically averaging the intervals (as they unfold in time sequence) and exploiting the last interval before the target events. The central tendency effect in temporal domain, similar to the central effect associated with other sensory properties such as weights and hues, is adaptively subject to the frame of reference (Hollingworth, 1910; Helson, 1947, 1948; Helson and Himelstein, 1955; Sherif et al., 1958; Thomas and Jones, 1962; Helson and Avant, 1967; Thomas et al., 1973; Hébert et al., 1974; Thomas and Strub, 1974; Newlin et al., 1978; Burr et al., 2013; Karaminis et al., 2016). Importantly, the temporal information near the target event is critical for crossmodal assimilation, wherein the recency effect prevails over the central tendency effect during the assimilation process (Burr et al., 2013; Karaminis et al., 2016). Crossmodal assimilation is more dependent on the temporal duration which entails the integration of task-relevant (temporal) information to be efficient within a short window (3 s) in addition to efficient working memory functions (Pöppel, 1997; Block and Gruber, 2014; Pöppel and Bao, 2014). However, the crossmodal assimilation is less subject to another process—attentional modulation (Talsma et al., 2010).

## Author contributions

YW conducted Experiment 1 and analyzed data. LC conducted Experiments 2–4, analyzed data and wrote the manuscript.

### Conflict of interest statement

The authors declare that the research was conducted in the absence of any commercial or financial relationships that could be construed as a potential conflict of interest.

## Supplementary material

Supplement 1 Demo of Ternus Display.

## References

[B1] Acerbi L. Wolpert D. M. Vijayakumar S. (2012). Internal representations of temporal statistics and feedback calibrate motor-sensory interval timing. PLoS Comput. Biol. 8:e1002771. 10.1371/journal.pcbi.1002771 23209386 PMC3510049

[B2] Allan L. G. (1974). Psychophysical theories of duration discrimination. Percept. Psychophys. 16, 26–34. 10.3758/BF03203244

[B3] Alsius A. Mottonen R. Sams M. E. Soto-Faraco S. Tiippana K. (2014). Effect of attentional load on audiovisual speech perception: evidence from ERPs. Front. Psychol. 5:727. 10.3389/fpsyg.2014.00727 25076922 PMC4097954

[B4] Alsius A. Navarra J. Campbell R. Soto-Faraco S. (2005). Audiovisual integration of speech falters under high attention demands. Curr. Biol. 15, 839–843. 10.1016/j.cub.2005.03.046 15886102

[B5] Block R. A. Gruber R. P. (2014). Time perception, attention, and memory: a selective review. Acta Psychol. 149, 129–133. 10.1016/j.actpsy.2013.11.003 24365036

[B6] Brainard D. H. (1997). The psychophysics toolbox. Spat. Vis. 10, 433–436. 10.1163/156856897X00357 9176952

[B7] Bruns P. Röder B. (2015). Sensory recalibration integrates information from the immediate and the cumulative past. Sci. Rep. 5:12739. 10.1038/srep12739 26238089 PMC4523860

[B8] Buchan J. N. Munhall K. G. (2011). The influence of selective attention to auditory and visual speech on the integration of audiovisual speech information. Perception 40, 1164–1182. 10.1068/p6939 22308887

[B9] Burr D. Della Rocca E. Morrone M. C. (2013). Contextual effects in interval-duration judgements in vision, audition and touch. Exp. Brain Res. 230, 87–98. 10.1007/s00221-013-3632-z 23864044

[B10] Cardinal L. J. (2015). Central tendency and variability in biological systems: part 2. J. Commun. Hosp. Intern. Med. Perspect. 5:28972. 10.3402/jchimp.v5.28972 26486117 PMC4612486

[B11] Chen L. Shi Z. Muller H. J. (2010). Influences of intra- and crossmodal grouping on visual and tactile Ternus apparent motion. Brain Res. 1354, 152–162. 10.1016/j.brainres.2010.07.064 20659437

[B12] Chen L. Vroomen J. (2013). Intersensory binding across space and time: a tutorial review. Atten. Percept. Psychophys. 75, 790–811. 10.3758/s13414-013-0475-4 23709064

[B13] Chen L. Zhang M. Ai F. Xie W. Meng X. (2016). Crossmodal synesthetic congruency improves visual timing in dyslexic children. Res. Dev. Disabil. 55, 14–26. 10.1016/j.ridd.2016.03.010 27022720

[B14] Chen Y. C. Spence C. (2017). Assessing the role of the ‘unity assumption' on multisensory integration: a review. Front. Psychol. 8:445. 10.3389/fpsyg.2017.00445 28408890 PMC5374162

[B15] Chuen L. Schutz M. (2016). The unity assumption facilitates cross-modal binding of musical, non-speech stimuli: the role of spectral and amplitude envelope cues. Atten. Percept. Psychophys. 78, 1512–1528. 10.3758/s13414-016-1088-5 27084701

[B16] Cohen M. A. Dennett D. C. Kanwisher N. (2016). What is the bandwidth of perceptual experience? Trends Cogn. Sci. 20, 324–335. 10.1016/j.tics.2016.03.006 27105668 PMC4898652

[B17] Cowan N. (2001). Metatheory of storage capacity limits. Behav. Brain Sci. 24, 154–176. 10.1017/S0140525X0161392X 11515286

[B18] Donohue S. E. Green J. J. Woldorff M. G. (2015). The effects of attention on the temporal integration of multisensory stimuli. Front. Integr. Neurosci. 9:32. 10.3389/fnint.2015.00032 25954167 PMC4407588

[B19] Donohue S. E. Roberts K. C. Grent-'t-Jong T. Woldorff M. G. (2011). The cross-modal spread of attention reveals differential constraints for the temporal and spatial linking of visual and auditory stimulus events. J. Neurosci. 31, 7982–7990. 10.1523/JNEUROSCI.5298-10.2011 21632920 PMC3224815

[B20] Duncan J. Martens S. Ward R. (1997). Restricted attentional capacity within but not between sensory modalities. Nature 387, 808–810. 9194561 10.1038/42947

[B21] Ernst M. Di Luca M. (2011). Multisensory perception: from integration to remapping, in Sensory Cue Integration, ed Trommershäuser J. (New York, NY: Oxford University Press), 225–250.

[B22] Friston K. (2010). The free-energy principle: a unified brain theory? Nat. Rev. Neurosci. 11, 127–138. 10.1038/nrn2787 20068583

[B23] Getty D. J. (1975). Discrimination of short temporal intervals: a comparison of two models. Percept. Psychophys. 18, 1–8. 10.3758/BF03199358

[B24] Grondin S. (1993). Duration discrimination of empty and filled intervals marked by auditory and visual signals. Percept. Psychophys. 54, 383–394. 10.3758/BF03205274 8414897

[B25] Gupta D. S. Chen L. (2016). Brain oscillations in perception, timing and action. Curr. Opin. Behav. Sci. 8, 161–166. 10.1016/j.cobeha.2016.02.021

[B26] Habets B. Bruns P. Roder B. (2017). Experience with crossmodal statistics reduces the sensitivity for audio-visual temporal asynchrony. Sci. Rep. 7:1486. 10.1038/s41598-017-01252-y 28469137 PMC5431144

[B27] Hébert J. A. Bullock M. Levitt L. Woodward K. G. Mcguirk F. D. (1974). Context and frequency effects in generalization of a human voluntary response. J. Exp. Psychol. 102, 456–462. 10.1037/h0035877 4815192

[B28] Helson H. (1947). Adaptation-level as frame of reference for prediction of psychophysical data. Am. J. Psychol. 60, 1–29. 10.2307/1417326 20288861

[B29] Helson H. (1948). Adaptation-level as a basis for a quantitative theory of frames of reference. Psychol. Rev. 55, 297–313. 10.1037/h0056721 18891200

[B30] Helson H. Avant L. L. (1967). Stimulus generalization as a function of contextual stimuli. J. Exp. Psychol. 73, 565–567. 10.1037/h0024319 6034011

[B31] Helson H. Himelstein P. (1955). A short method for calculating the adaptation-level for absolute and comparative rating judgments. Am. J. Psychol. 68, 631–637. 10.2307/1418791 13275609

[B32] Hillock A. R. Powers A. R. Wallace M. T. (2011). Binding of sights and sounds: age-related changes in multisensory temporal processing. Neuropsychologia 49, 461–467. 10.1016/j.neuropsychologia.2010.11.041 21134385 PMC3140703

[B33] Hillock-Dunn A. Grantham D. W. Wallace M. T. (2016). The temporal binding window for audiovisual speech: children are like little adults. Neuropsychologia 88, 74–82. 10.1016/j.neuropsychologia.2016.02.017 26920938

[B34] Hollingworth H. L. (1910). The central tendency of judgment. J. Philos. Psychol. Sci. Methods 7, 461–469. 10.2307/2012819

[B35] Jazayeri M. Shadlen M. N. (2010). Temporal context calibrates interval timing. Nat. Neurosci. 13, 1020–1026. 10.1038/nn.2590 20581842 PMC2916084

[B36] Jones M. R. McAuley J. D. (2005). Time judgments in global temporal contexts. Percept. Psychophys. 67, 398–417. 10.3758/BF03193320 16119390

[B37] Karaminis T. Cicchini G. M. Neil L. Cappagli G. Aagten-Murphy D. Burr D.. (2016). Central tendency effects in time interval reproduction in autism. Sci. Rep. 6:28570. 10.1038/srep28570 27349722 PMC4923867

[B38] Keetels M. Vroomen J. (2008). Tactile–visual temporal ventriloquism: no effect of spatial disparity. Percept. Psychophys. 70, 765–771. 10.3758/PP.70.5.765 18613625

[B39] Kleiner M. Brainard D. Pelli D. Ingling A. Murray R. Broussard C. (2007). What's new in psychtoolbox-3. Perception 36, 1–16. 10.1068/v070821

[B40] Klemen J. Buchel C. Rose M. (2009). Perceptual load interacts with stimulus processing across sensory modalities. Eur. J. Neurosci. 29, 2426–2434. 10.1111/j.1460-9568.2009.06774.x 19490081

[B41] Klemen J. Chambers C. D. (2012). Current perspectives and methods in studying neural mechanisms of multisensory interactions. Neurosci. Biobehav. Rev. 36, 111–133. 10.1016/j.neubiorev.2011.04.015 21569794

[B42] Lewkowicz D. J. Flom R. (2014). The audiovisual temporal binding window narrows in early childhood. Child Dev. 85, 685–694. 10.1111/cdev.12142 23888869 PMC3954953

[B43] Li Y. Long J. Huang B. Yu T. Wu W. Li P.. (2016). Selective audiovisual semantic integration enabled by feature-selective attention. Sci. Rep. 6:18914. 10.1038/srep18914 26759193 PMC4725371

[B44] Mégevand P. Molholm S. Nayak A. Foxe J. J. (2013). Recalibration of the multisensory temporal window of integration results from changing task demands. PLoS ONE 8:e71608. 10.1371/journal.pone.0071608 23951203 PMC3738519

[B45] Meredith M. A. Nemitz J. W. Stein B. E. (1987). Determinants of multisensory integration in superior colliculus neurons. *I.* Temporal Factors J. Neurosci. 7, 3215–3229. 3668625 10.1523/JNEUROSCI.07-10-03215.1987 PMC6569162

[B46] Miller J. E. Carlson L. A. McAuley J. D. (2013). When what you hear influences when you see: listening to an auditory rhythm influences the temporal allocation of visual attention. Psychol. Sci. 24, 11–18. 10.1177/0956797612446707 23160202

[B47] Misceo G. F. Taylor N. J. (2011). A “unity assumption” does not promote intersensory integration. Exp. Psychol. 58, 385–390. 10.1027/1618-3169/a000107 21592944

[B48] Nakajima Y. ten Hoopen G. Hilkhuysen G. Sasaki T. (1992). Time-shrinking: a discontinuity in the perception of auditory temporal patterns. Percept. Psychophys. 51, 504–507. 10.3758/BF03211646 1594440

[B49] Newlin R. J. Rodgers J. P. Dickson J. F. Strub H. Thomas D. R. (1978). The central tendency effect in stimulus generalization: effects of establishing a “preexperimental” frome of reference. Percept. Psychophys. 24, 161–167. 10.3758/BF03199543 693251

[B50] Parise C. V. Spence C. Ernst M. O. (2012). When correlation implies causation in multisensory integration. Curr. Biol. 22, 46–49. 10.1016/j.cub.2011.11.039 22177899

[B51] Penney T. B. Gibbon J. Meck W. H. (2000). Differential effects of auditory and visual signals on clock speed and temporal memory. J. Exp. Psychol. Hum. Percept. Perform. 26, 1770–1787. 10.1037/0096-1523.26.6.1770 11129373

[B52] Pöppel E. (1997). A hierarchical model of temporal perception. Trends Cogn. Sci. 1, 56–61. 10.1016/S1364-6613(97)01008-5 21223864

[B53] Pöppel E. Bao Y. (2014). Temporal windows as a bridge from objective to subjective time, in Subjective Time, eds Lloyd D. Arstila V. (Cambridge, MA: MIT Press), 241–261.

[B54] Powers A. R. Hevey M. A. Wallace M. T. (2012). Neural correlates of multisensory perceptual learning. J. Neurosci. 32, 6263–6274. 10.1523/JNEUROSCI.6138-11.2012 22553032 PMC3366559

[B55] Powers A. R. Hillock A. R. Wallace M. T. (2009). Perceptual training narrows the temporal window of multisensory binding. J. Neurosci. 29, 12265–12274. 10.1523/JNEUROSCI.3501-09.2009 19793985 PMC2771316

[B56] Rammsayer T. H. (2010). Differences in duration discrimination of filled and empty auditory intervals as a function of base duration. Atten. Percept. Psychophys. 72, 1591–1600. 10.3758/APP.72.6.1591 20675803

[B57] Rammsayer T. H. Lima S. D. (1991). Duration discrimination of filled and empty auditory intervals: cognitive and perceptual factors. Percept. Psychophys. 50, 565–574. 10.3758/BF03207541 1780204

[B58] Roach N. W. McGraw P. V. Whitaker D. J. Heron J. (2017). Generalization of prior information for rapid Bayesian time estimation. Proc. Natl. Acad. Sci. U.S.A. 114, 412–417. 10.1073/pnas.1610706114 28007982 PMC5240697

[B59] Sherif M. Taub D. Hovland C. I. (1958). Assimilation and contrast effects of anchoring stimuli on judgments. J. Exp. Psychol. 55, 150–155. 10.1037/h0048784 13513928

[B60] Shi Z. Burr D. (2016). Predictive coding of multisensory timing. Curr. Opin. Behav. Sci. 8, 200–206. 10.1016/j.cobeha.2016.02.014 27695705 PMC5040498

[B61] Shi Z. Chen L. Muller H. J. (2010). Auditory temporal modulation of the visual Ternus effect: the influence of time interval. Exp. Brain Res. 203, 723–735. 10.1007/s00221-010-2286-3 20473749

[B62] Shi Z. Church R. M. Meck W. H. (2013). Bayesian optimization of time perception. Trends Cogn. Sci. 17, 556–564. 10.1016/j.tics.2013.09.009 24139486

[B63] Stevenson R. A. Siemann J. K. Schneider B. C. Eberly H. E. Woynaroski T. G. Camarata S. M.. (2014). Multisensory temporal integration in autism spectrum disorders. J. Neurosci. 34, 691–697. 10.1523/JNEUROSCI.3615-13.2014 24431427 PMC3891950

[B64] Stevenson R. A. Zemtsov R. K. Wallace M. T. (2012). Individual differences in the multisensory temporal binding window predict susceptibility to audiovisual illusions. J. Exp. Psychol. Hum. Percept. Perform. 38, 1517–1529. 10.1037/a0027339 22390292 PMC3795069

[B65] Sugano Y. Keetels M. Vroomen J. (2010). Adaptation to motor-visual and motor-auditory temporal lags transfer across modalities. Exp. Brain Res. 201, 393–399. 10.1007/s00221-009-2047-3 19851760 PMC2832876

[B66] Sugano Y. Keetels M. Vroomen J. (2012). The Build-up and transfer of sensorimotor temporal recalibration measured via a synchronization task. Front. Psychol. 3:246. 10.3389/fpsyg.2012.00246 22807921 PMC3395050

[B67] Sugano Y. Keetels M. Vroomen J. (2016). Auditory dominance in motor-sensory temporal recalibration. Exp. Brain Res. 234, 1249–1262. 10.1007/s00221-015-4497-0 26610349 PMC4828498

[B68] Talsma D. Doty T. J. Woldorff M. G. (2007). Selective attention and audiovisual integration: is attending to both modalities a prerequisite for early integration? Cereb. Cortex 17, 679–690. 10.1093/cercor/bhk016 16707740

[B69] Talsma D. Senkowski D. Soto-Faraco S. Woldorff M. G. (2010). The multifaceted interplay between attention and multisensory integration. Trends Cogn. Sci. 14, 400–410. 10.1016/j.tics.2010.06.008 20675182 PMC3306770

[B70] Tang X. Wu J. Shen Y. (2016). The interactions of multisensory integration with endogenous and exogenous attention. Neurosci. Biobehav. Rev. 61, 208–224. 10.1016/j.neubiorev.2015.11.002 26546734 PMC4753360

[B71] Ternus J. (1926). Experimentelle untersuchungen über phänomenale identität. Psychol. Forsch. 7, 81–136. 10.1007/BF02424350

[B72] Thomas D. R. Jones C. G. (1962). Stimulus generalization as a function of the frame of reference. J. Exp. Psychol. 64, 77–80. 10.1037/h0043304 13920759

[B73] Thomas D. R. Strub H. (1974). Adaptation level and the central tendency effect in stimulus generalization. J. Exp. Psychol. 103, 466–474. 10.1037/h0037184

[B74] Thomas D. R. Svinicki M. D. Vogt J. (1973). Adaptation level as a factor in human discrimination learning and stimulus generalization. J. Exp. Psychol. 97, 210–219. 10.1037/h0033908

[B75] Treutwein B. Strasburger H. (1999). Fitting the psychometric function. Percept. Psychophys. 61, 87–106. 10.3758/BF03211951 10070202

[B76] Vatakis A. Spence C. (2007). Crossmodal binding: evaluating the “unity assumption” using audiovisual speech stimuli. Percept. Psychophys. 69, 744–756. 10.3758/BF03193776 17929697

[B77] Vatakis A. Spence C. (2008). Evaluating the influence of the ‘unity assumption' on the temporal perception of realistic audiovisual stimuli. Acta Psychol. 127, 12–23. 10.1016/j.actpsy.2006.12.002 17258164

[B78] Vroomen J. Bertelson P. de Gelder B. (2001). The ventriloquist effect does not depend on the direction of automatic visual attention. Percept. Psychophys. 63, 651–659. 10.3758/BF03194427 11436735

[B79] Vroomen J. Keetels M. (2010). Perception of intersensory synchrony: a tutorial review. Atten. Percept. Psychophys. 72, 871–884. 10.3758/APP.72.4.871 20436185

[B80] Wahn B. Konig P. (2015). Audition and vision share spatial attentional resources, yet attentional load does not disrupt audiovisual integration. Front. Psychol. 6:1084 10.3389/fpsyg.2015.01084 26284008 PMC4518141

[B81] Wallace M. T. Stevenson R. A. (2014). The construct of the multisensory temporal binding window and its dysregulation in developmental disabilities. Neuropsychologia 64, 105–123. 10.1016/j.neuropsychologia.2014.08.005 25128432 PMC4326640

[B82] Wichmann F. A. Hill N. J. (2001). The psychometric function: I. fitting, sampling, and goodness of fit. Percept. Psychophys. 63, 1293–1313. 10.3758/BF03194544 11800458

