# Supplementary material for: Temporal Reference, Attentional Modulation, and Crossmodal Assimilation
Source: Front Comput Neurosci. 2018 Jun 5;12:39. doi: 10.3389/fncom.2018.00039 (PMC5996128; doi:10.3389/fncom.2018.00039)
Supplement: Supplement 1 — Demo of Ternus Display. [file Presentation_1.pptx]

## Slide 1
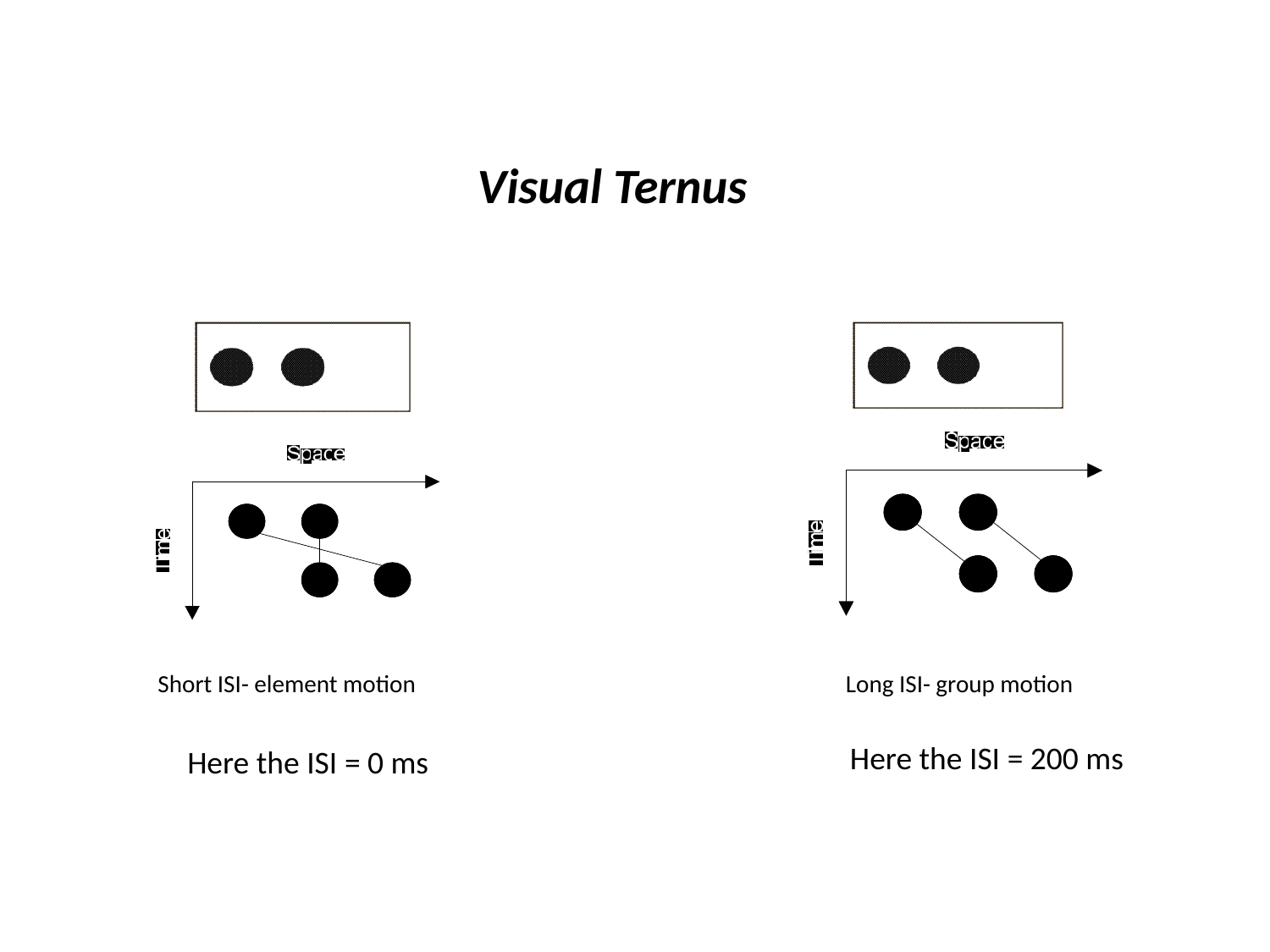

Visual Ternus
Short ISI- element motion
Long ISI- group motion
Here the ISI = 200 ms
Here the ISI = 0 ms
